# Does Playing Video Games Before Bedtime Affect Sleep?

**DOI:** 10.7759/cureus.4977

**Published:** 2019-06-23

**Authors:** Jeffrey A Miskoff, Moiuz Chaudhri, Benjamin Miskoff

**Affiliations:** 1 Internal Medicine, Jersey Shore University Medical Center, Neptune City, USA

**Keywords:** poor sleep, sleep duration, sleep quality, pediatrics, video games, sleep disorders, rem behavior disorder

## Abstract

Sleep serves a vital role in our ability to function on a daily basis and may be affected by various activities such as playing video games. Teenagers are one of the largest consumers of video games and if played before bedtime may lead to the release of certain neurotransmitters which may, in turn, alter sleep architecture and reduce sleep efficiency. The purpose of this study is to measure 1) sleep efficiency 2) sleep latency 3) time spent in rapid eye movement (REM) stage with and without playing video games 30 minutes to 60 minutes before bedtime. For this study, one patient was recruited. The study was completed using a television, video game console, and a video game (Red Dead Redemption 4), Apnea Risk Evaluation System (ARES) nocturnal polysomnogram (NPSG) unit, a bed and a blanket situated in a quiet room, a computer, printer, and a notebook for data recording. REM time and sleep latency were also measured. There were 45.6 minutes of REM with video games and 56.4 minutes of REM without video games. This was equivalent to 13.06% and 15.74% of the total sleep time, respectively. The sleep latency with video games was shorter than without video games (11.4 and 23 minutes, respectively). Result suggests that there is no significant difference in sleep efficiency with and without video games. However, sleep latency decreased, and REM increased with video games.

## Introduction

Sedentary lifestyle and poor sleep have been linked to a myriad of morbidity creating an enormous burden felt by the individual and the economy as a whole [1-2]. This epidemic is more pronounced in our youth as they engage in playing video games and other technology-driven activities. Impact of these sedentary activities can be measured by studying the quality and quantity of sleep. Poor sleep can decrease a person's day to day functionality and if maintained can lead to various health and behavior problems [3-4].

Video games are a part of our culture, as illustrated by its place in the entertainment industry. Video games rank as one of the largest entertainment industries in the United States [5]. According to the report, about 155 million people play video games translating into nearly $22 billion deposited into our economy. Video games before bedtime have been thought to produce poor sleep, especially sleep latency [6-7].

Although all age groups enjoy playing video games, the gaming industry is dominated by teenagers. Video games before bedtime have been associated with poor sleep, but the evidence supporting this is less than robust. Purpose of this study was to investigate this association and to provide some insight into video games and its impact on the quality and quantity of sleep. For this study, the primary endpoint was sleep efficiency (number of minutes spent sleeping/total number of minutes spent in bed). In addition, secondary endpoints consisted of rapid eye movement (REM) and sleep latency (amount of time it takes to go from wakefulness to entering sleep).

## Case presentation

This study included one patient, and it was designed to be completed in 10 consecutive nights. For the first five nights, the patient played a video game for one hour immediately before going to sleep. The second set of five nights, the patient meditated for one hour before going to bed instead of playing video games. This study was completed using a television, video game console and video game (Red Dead Redemption 4), Apnea Risk Evaluation System (ARES) nocturnal polysomnogram (NPSG) unit, a bed and a blanket situated in a quiet room, a computer, printer, and a notebook for data recording. Furthermore, the patient was required to follow the following steps to ensure study integrity. Primary and secondary outcomes of this study were evaluated using paired sample t-test, and the p-value was determined.

Step 1: Control outside daily activity by doing the same thing every day, control stress by taking breaks throughout the day, control food intake by eating the same thing every day, and control time awake by going to sleep at the same time and waking up at the same time every day.

Step 2: On night 1, 2, 3, 4 and 5, play one hour of video games from 10:30 pm to 11:30 pm.

Step 3: Prepare for bed and place the ARES device from 11:30 pm to 12:00 am.

Step 4: Lights off at midnight and record on sleep diary. Activate device and wear until rising time. Turn off the device in the morning and recharge as needed.

Step 5: Record sleep efficiency and along with other secondary outcomes.

Step 6: On night 6, 7, 8, 9 and 10, meditate or perform a low-level activity for one hour from 10:30 pm to 11:30 pm and then repeat steps 3 to 5.

Step 7: Record on a sleep diary bedtime, rise time, and other activity.

Mean sleep efficiency was calculated to be 85.76% and 87.69% with and without video games respectively with a p-value of 0.507. Mean REM sleep was calculated to be 45.6 minutes and 56.4 minutes with and without video games respectively with a p-value of 0.30. Mean sleep latency was calculated to be 11.4 minutes and 23.0 minutes with and without video games respectively and a p-value of 0.19 (Table 1).

**Table 1:** Summary of primary endpoints REM: rapid eye movement.

	Mean sleep efficiency (%)	Mean sleep latency (minutes)	Mean REM (minutes)
Video games	85.76	11.4	45.6
Without video games	87.69	23.0	56.4
P-values	0.507	0.19	0.30

Mean REM sleep as a percentage was calculated to be 13.0% and 14.4% with and without video games respectively with a p-value of 0.378 (Tables 2-3).

**Table 2:** REM sleep with video games REM: rapid eye movement.

	Night (number)	REM (minutes)	REM (%)
Video games			
	1	48	12.9
	2	36	10
	3	42	12.2
	4	30	8.7
	5	72	21.5
Mean		45.6	13.0

**Table 3:** REM sleep without video games REM: rapid eye movement.

	Night (number)	REM (minutes)	REM (%)
No video games			
	6	36	9.7
	7	66	66
	8	72	72
	9	60	60
	10	48	48
Mean		51	14.4

## Discussion

Sleep is one of the most important and fundamental components of human existence for several reasons. Each stage of sleep serves a different purpose ranging from the initial relaxation to experiencing a transient state of muscle paralysis in Stage 1 and Stage 5 (REM), respectively. The sleep-wakefulness cycle is a complex process requiring precise interaction between different neurotransmitters. Monoamines (dopamine, norepinephrine, and serotonin), acetylcholine, histamine, orexin are associated with wakefulness while adenosine, neurotransmitter γ-aminobutyric acid (GABA), melatonin and galanin promote sleep. Although various neurotransmitters are important in this complex process, acetylcholine serves as an integral modulator in both states, wakefulness, and sleep, especially REM [8-9]. REM sleep allows processing and integration of newly acquired information into long term storage along with improving human performance across a variety of tasks [10-11].

Although video games are very entertaining to many individuals young and older, their adverse association with other aspects of life should not be ignored. Data suggests that video gamers are more likely to be obese than individuals who do not play video games [12]. Numerous studies have reported that engaging in video games before bedtime are associated with poor sleep. A study by Weaver et al. evaluated the effects of video games on sleep latency, an average number of hours, and its impact on their quality of day to day functionality. Their result suggests that video games affect the quality of life but not as severe as originally thought [6].

In this study, we attempted to provide additional information regarding video games and their impact on sleep. We found that sleep efficiency decreased when the participant played video games (sleep efficiency = 85.76%) immediately before going to sleep instead of taking part in other non-stimulant activity (sleep efficiency = 87.6%). Literature provides substantial anecdotal and evidence-driven data linking video games with poor sleep [6,9,13]. Furthermore, studies have investigated this association by focusing on the type of video games citing that violent games carry a higher negative burden. According to our data, the participant had shorter REM sleep after playing video games, and it is on par with published literature [14-15]. In a recent study, 16 male patients were analyzed for sleep associated deficiencies after playing video games for one night. The authors concluded that these individuals exhibited poor attention and focus related to video games; these deficiencies were pronounced after playing video games on consecutive nights [16]. Literature suggests that the impact of video games on sleep can manifest in different ways including day to day functionality, emotional, psychological, and behavioral dysfunction [13,17]. Furthermore, video games can lead to delayed development of neuronal microstructure network which can impact verbal intelligence of the developing brain along with emotional dysfunction [18]. Moreover, they concluded that the duration of video gameplay along with the type of video game being played is another important factor which needs further investigation [18-19].

This study’s limitations stem from small sample size which decreased statistical power and not allowing to reach statistical and clinically significant findings. Although some data suggested the negative effect of video games on sleep, conclusive results were not reached.

## Conclusions

Sleep efficiency (primary outcome) was not significantly affected by playing video games before bedtime. However, some trends were noted with the secondary outcomes of REM time and sleep latency. Mean REM time and sleep latency were decreased after playing video games, however, the difference is not statistically significant. In addition, a definitive conclusion cannot be made regarding the effect of playing video games on these two variables due to the small sample size. Therefore, further studies with large sample size are needed to reach a definitive conclusion.
